# Mettauer on the Use of the Unripe Persimmon, or Diospyros Virginiana, as a Therapeutic Agent

**Published:** 1842-10-15

**Authors:** 


### ANALECTA.

*On the use of the Unripe Fruit of the Persimmon, or Diospyros Virginiana, as a Therapeutic Agent.*—Dr. J. P. METTAUER, of Prince Edward Court

House, Va., in the October number of the American Journal of the Medical Sciences, calls the attention of the profession to this article. He says:

"Our first trial consisted of the use of the simple infusion, formed by pouring a teacup of boiling water upon half a dozen of the half-grown persimmons slightly crushed. As soon as the infusion was cool, we directed a tea-spoonful of it to be given to an infant rather more than a year old, sweetened with refined sugar, every second hour, until the watery discharges from the bowels, under which it was rapidly wasting, should be arrested, or the infusion be found to disagree. This experiment was most satisfactory, and the result truly gratifying. The persimmon had only been administered twice before the bowels were restrained; and after the third dose it was suspended, until an alvine discharge could be procured by an enema. After this, the remedy was only administered occasionally, as the diarrhœa threatened to recur, alternating with it from time to time enemata, or mild internal aperients. In a fortnight after this agent was first administered, the child was able to run about the house, and very soon recovered perfectly.

Since the first trial with this new remedy, we have had many opportunities for using it in similar cases, and even in common diarrhœa, and the results have uniformly been decidedly favourable and salutary.

After various experiments and trials with this substance, we have adopted four standard preparations for using it; that is, the tea or infusion; the syrup, the vinous and acetous tinctures.

The infusion is a very active and efficient form, and will be found both agreeable and convenient for administration: it can only be employed, however, during the season which affords the fruit, and for this reason only, it is to be regarded, perhaps, as the least unexceptionable of the preparations of this article. Nevertheless, the infusion may be used with great advantage during the summer and early autumnal months—which period of the year is most prolific of the forms of diarrhœa in which astringents are allowable. It may be prepared by infusing from one to two ounces of the fresh immature fruit slightly crushed, in a common teacup of boiling water; and of the cool infusion sweetened with refined sugar, from one to three teaspoonfuls may be given to infants once an hour, or after long intervals, until the restraining effect is produced. Occasionally the tea may be rendered aromatic by adding cassia bark, pimento, ginger and the like; or it may be animated with French brandy, gin, or wine to render it more palatable. When to be used with adults the doses must be augmented to from one to three table-spoonfuls; and given after the intervals already stated.

The syrup may be prepared by converting the infusion already described into a syrup, by adding to it refined sugar, and gently boiling them down to a proper consistency for keeping. This is decidedly the most convenient and useful form of using the persimmon; it is also the most agreeable, as the sugar greatly modifies the rough taste. This syrup may be variously combined for administration, and is ready and at home at all seasons of the year. It also possesses the astringency of the persimmon in great purity, and will retain it for an indefinite period of time without the least deterioration.

In preparing the syrup, care should be taken not to urge the process of converting the infusion into a syrup too rapidly; gentle and gradual boiling answers best; indeed, the fluid should just be kept to the boiling point; and the process must be conducted over a sand bath, or a salt-water bath; and glass vessels should invariably be used for the purpose.

For infants, the doses of the syrup may be very nearly as we have advised of the infusion; perhaps they should be somewhat smaller. With adults we have invariably used from two to four tea-spoonfuls to the dose.

The vinous tincture may be prepared by digesting one pound of the green persimmon recently procured and a little crushed, in one pint and a half of port, or any other wine, exposed daily to the solar heat for fourteen days. After this the tincture may be filtered for use. This is an elegant preparation, and possesses the astringent powers of the persimmon in great purity, and in a most convenient form for administration. It is not, however, applicable to every variety of diarrhœa, by reason of the stimulating menstruum used in its preparation. This preparation is more especially applicable to the treatment of adult cases of diarrhœa, though we have occasionally employed it with infants likewise. The dose for adults is very nearly the same as advised of the syrup. With infants the dose should rarely exceed a tea-spoonful, and must invariably be small to commence with, say not more than one-third of a tea-spoonful; and the medicine should always be sweetened with refined sugar.

The acetous tincture is prepared by digesting two pounds of the recent fruit a little crushed, in two pints of strong pure apple-vinegar, fourteen days exposed to the solar heat; the tincture may then be filtered for use. This preparation is chiefly designed for external use, especially for gargles and cataplasms. It is most valuable in tonsillary affections, especially when they follow scarlatina, or chronic catarrh, used as a gargle. It is also exceedingly useful as a cataplasm in whitlow, or inflammation of the mammæ threatening milk abscess, To form it into a cataplasm, it will only be necessary to convert any quantity of the tincture into a poultice, by uniting with it when hot, the requisite proportions of any kind of farinaceous material; even the fruit may be used. We have also employed it in the early stages of dysentery after a brisk cathartic, and with decidedly beneficial effects: in this disease it promises to be eminently useful. It is a more efficient remedy in this disease than the solution of common salt in vinegar, to which, however, it assimilates itself very much in its remedial action.

The astringency of the persimmon is peculiarly adapted to the treatment of every form of diarrhœa. In the diarrhœa of infancy, we have often used the infusion and syrup with distinguished benefit. The tinctures we have also occasionally employed, but not with very decided benefit with these tender subjects, chiefly, we think, because we were unwilling to hazard their use with them in commanding doses, We have frequently united the infusion and syrup, with the syrups of rhubarb and senna, or the infusion of senna; and occasionally with calomel, with benefit. With stimulants and tonics these preparations may also often be associated with advantage. In some exceedingly bad cases of protracted Mississippi diarrhœa, we have used the persimmon with the most triumphant and salutary effects. In these cases we generally premise one or two doses of blue mercurial powder, about twelve grains, before commencing with the persimmon; and the infusion, syrup, or vinous tincture given in port wine, one of the forms of using it generally adopted by us. Should the remedy restrain the bowels very suddenly, aperients must be interposed; and for this purpose, nothing answers so well as the syrup of rhubarb, or the compound syrup of senna.

Occasionally we have found it useful to combine grain portions of ipecacuanha with the persimmon, with a view to its diaphoretic effects; with adults, especially, it will be found exceedingly beneficial to use such a combination.

In the chronic stages of dysentery, we have employed these forms of the persimmon very beneficially. When used in this disease, they must be united with the syrup of rhubarb, or senna; or, combined with the oleaginous emulsion; and generally, paregoric in sufficient doses to impress the system decidedly, should be given with them at night.

In uterine hemorrhage, and especially in menorrhagia, we have employed the infusion, both as an internal remedy, and by way of injection, per vaginam, with great benefit. In these fluxes it is destined to be eminently useful from the promptness and great potency of its action as an astringent; much, however, remains to be known of the remedial operation and applicability of the persimmon as a therapeutic agent.

